# Electroactive polymer gels as probabilistic reservoir automata for computation

**DOI:** 10.1016/j.isci.2022.105558

**Published:** 2022-11-14

**Authors:** Vincent Strong, William Holderbaum, Yoshikatsu Hayashi

**Affiliations:** 1 Department of Biomedical Sciences and Biomedical Engineering, School of Biological Sciences, University of Reading, Reading, Berkshire RG67BE, UK

**Keywords:** Theoretical physics, Materials science, Polymers

## Abstract

Electroactive Polymer (EAP) hydrogels are an active matter material used as actuators in soft robotics. Hydrogels exhibit active matter behavior through a form of memory and can be used to embody memory systems such as automata. This study exploited EAP responses, finding that EAP memory functions could be utilized for automaton and reservoir computing frameworks. Under sequential electrical stimulation, the mechanical responses of EAPs were represented in a probabilistic Moore automaton framework and expanded through shaping the reservoir’s energy landscape. The EAP automaton reservoir’s computational ability was compared with digital computation to assess EAPs as computational resources. We found that the computation in the EAP’s reaction to stimuli can be presented through automaton structures, revealing a potential bridge between EAP’s use as an integrated actuator and controller, i.e., our automaton framework could potentially lead to control systems wherein the computation was embedded into the media dynamical responses.

## Highlights

• EAP gel memory mechanics were demonstrated via voltage potential measurements
• Probabilistic Moore automata were constructed from EAP gel responses to stimulation
• Through tuning response encoding a computational reservoir was created
• The reservoir was shown as more memory efficient than general digital alternatives

## Graphical abstract

**Figure:**
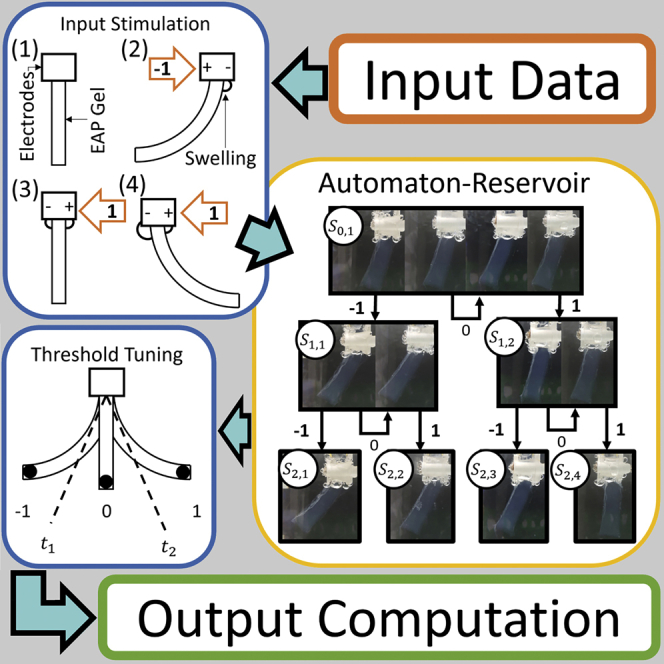

## Introduction

Computation spans many fields of study from algorithmic mathematics to hardware design and biological simulation.^1^ In computational systems, the hardware and information processing dictate each other’s requirements. Traditional computational systems separate the physical hardware and software side (implementation of algorithms) to allow for a diverse range of operations within the same architecture.^2^

However, some computational approaches combine the physical and algorithmic components to create a system that is tailored to a specific task. For example, morphological computing explores this integration of computing with the physical body, and proposes that control aspects can be outsourced to the body as the functions are already “encoded” within.^3^ Through this encoding, morphological computing allows a system to include the environment in its processes because the physical body is influenced by the environment the process is in turn influenced. By outsourcing control to the body, the system becomes more open to environmental effects in a way that can be supportive of the control instead of something to resist. This embodiment of the environment in a control system is conceptualized in the idea of embodied cognition. The idea of embodied cognition, originating in psychology, is that the body’s interactions with the environment constitute or contribute to cognition, meaning mental processes are not only computational processes.^4^ An agent’s cognition or computation is strongly influenced by aspects of an agent’s body.^5^ Cognition arises from bodily interactions with the world and depends on the kinds of experiences that come from having a particular body.^6^ For both concepts of morphological computing and embodied cognition the bidirectional influence is exploitable. A stimulated body material acts as a computational unit causing a change in the body and influencing actions on future stimulations. For example, a single material can potentially contain both the cognition and actuation elements, but requires bodies with highly complex dynamics to be computationally powerful.^7^^,^^8^ Self-oscillating hydrogels have been explored as a form of self-regulating repeating actuator^9^ combining control and actuation in a single body. However, these actuators had little to no external control and could not react to changes in the environment. Through morphological and embodied theories, changing environments can be reflected in a body’s behavior. Research has been carried out that utilizes the inherent properties of a soft arm in its control.^10^ In this example, the compliance of the arm was used as a component for its own control. The way in which the arm deformed in reaction to actuation was used to alter further actuations, creating a feedback loop where the soft-body’s response dictated future behavior. There have been many other explorations into combining morphological concepts with robotics including those inspired by plant root behavior. In this example, a compliant body allows a robot to find the most efficient route through soil without external control.^11^^,^^12^ A form of universal gripper was developed that used a compliant bag that conformed to a surface shape then applied pressure to grip. This technology allowed complex shapes to be grasped without the need to calculate gripping trajectories as all ’calculations’ were done by the compliance of the surface.^13^ Each of these examples uses inherent properties of the physical body to stand in for control that would normally be handled externally. So, by exploring materials that contain highly complex dynamics, is it possible to combine both computation and actuation in a single medium allowing for the actuator to, in part, control itself.

To investigate this question a single medium with highly complex dynamics is needed, one that demonstrates the control concepts of morphological computing and embodied cognition in its reaction to stimuli. Active matter is a field of study into materials that present such behaviors. Active matter materials are composed of many active agents which consume energy to drive mechanical forces. There are many forms of active matter from active fluids or soft robotics.^14^ Importantly these active agents, although independent, influence each other leading to a form of parallel computation, a feature that has been exploited in some chemical processor systems utilizing Belousov–Zhabotinsky (BZ) reactions.^15^^,^^16^

However, most computational systems require memory to process functions. Finite automata use the memory of their current state to perform computations, morphological computation media are no different. In such morphological active matter systems, functions of memory are manifested through the distribution of active agents and their interactions with each other.^17^^,^^18^ Although individually the agents are simple and memory-less, together they can embody a response to stimuli. Stimulation changes the active agent’s contribution to the system by changing local concentrations, velocities, and other properties. When given additional stimuli these collective properties, among the many active agents, alter the response. Each consecutive response to stimuli is influenced by previous responses.

The way in which active matter manifests memory in this fashion has also been employed in chemical systems and exploited to perform computation as automata.^19^ Another physical chemistry system that displays a memory function is active hydrogels.^20^ In active hydrogels, free floating ions act as the active agents. Through the application of electric fields, the ions can be influenced to move causing changes in the polymer structure.^21^ A hysteresis is induced by changes in the polymer structure and causes subsequent stimulations to generate less actuation.^22^ This hysteresis means, as with other active matter systems, previous responses to stimuli affect future responses leading to a memory function.

These morphological active matter examples present memory-based behavior in their actuation. However, to investigate if it is possible to combine actuation and control, a computational structure is needed that these behaviors can be exploited through. The most common memory based computational structure is that of the automata, indeed automata exist throughout nature morphologically and nature initially inspired their design.^23^ Automata are a computational structure that process information in sequence according to a set of programmed rules.^24^ Information in and out of the automaton must follow a language definition, comprised of a set of defined symbols (or words) with defined grammar to form a sequence (or sentence). The language dictates what the automaton accepts as input and what the automaton gives as output. Output can use an entirely separate language to the input and be as simple as acceptance or rejection of certain inputs.

The simplest automaton structures are deterministic, however active matter systems in practice are non-deterministic as each input can lead to several outcomes. However, even highly dynamic systems can be simplified to sets of rules given a large enough rule set.^25^ Probabilistic automata are a generalization of non-deterministic finite automaton that traverse states based on weighted state transitions.^26^^,^^27^ Because of their generalization, probabilistic automata can be applied to a significant number of applications and are often used in statistical modeling.^28^ The probabilistic automaton structure allows large complex systems to be simplified into state machines, where probability distributions are converted to weighted state transitions.^29^ These automata fit real world behavior of complex bodies such as active matter systems but do not traditionally take inputs. Without the ability to take inputs a system cannot be used computationally. However, Moore Machine automata are a type of finite-state transducer (FST) automata^30^ that traverse states based on sequential inputs, giving an output at each state. Through the combination of behavioral features in these automata frameworks, active matter and other such morphological systems could be represented in a computational context as a probabilistic Moore automaton.

The probabilistic Moore automaton structure, when applied to the EAP hydrogel, offers many advantages over the more conventional computational structures such as Moore machine. Moore machine automaton are most often used to model computer programs and computational logic,^31^ this means that by using them as a foundation any framework generated already has a strong base in computational logic allowing for easier implementation within applications. However, without the probabilistic element the full behavior of the EAP hydrogel is restricted to deterministic approximations, this obstructs an entire region of complexity that could be utilized for computation. Probabilistic computing, or stochastic computing, has been a field of interest since the 1960s, allowing for more efficient computation with high error tolerance^32^ and allowing for broader ranges of computations.^33^ Using the conventional computing techniques of Moore machine automata as a base gives a head start in framework development through the wealth of knowledge in the field of automata computing. In addition, this allows the final framework to be more easily applied to problems currently being approached via automaton based solutions. Building the probabilistic elements on top of the Moore machine automata allows for faster implementation, while also allowing the full computational potential of the medium to be exploited. Probabilistic elements can provide a broader computational landscape, when compared to more convectional computational structures. Although these automaton frameworks provide a flexible and robust computation scheme, the true potential lies in exploiting the inherent parallel processing of active matter.^19^ Neural networks are an inherently parallel computational construct but are typically implemented in software or specially designed hardware; reservoir computing expands this implementation dependency.

Reservoir computing is inspired by Recurrent Neural Network (RNN) frameworks where the dynamics of a fixed non-linear system, called a reservoir, is used as part of a neural network to map input and output signals to higher dimensional space.^34^ The reservoir feeds into a layer of neurons known as the output layer. The weights of these output neurons are tuned to achieve learning in the system much like in standard RNNs. The reservoir can be any kind of medium that can encode temporal problems into higher dimensions to generate recurrent connections between data.^35^ For example, water ripples in a bucket have been used as a reservoir to encode image data for pattern recognition.^36^ In addition, a length of optical fiber has been used to introduce a time delay and gain in feedback to create a reservoir for use in speech recognition.^37^

As with morphological computing, the key use of reservoir computing systems is to exploit alternative computation media to solve problems that would otherwise be inefficient for traditionalcomputing.^35^ In automaton theory the automaton is designed to accomplish a specific task through the definition of its language.^24^ Automaton structures are limited by the language that they use. However, using reservoir computing as an additional layer that alters the automaton language to suit a given task could expand the capabilities of the system. Given the morphological nature of reservoir computing, can reservoir computing be combined with probabilistic Moore automata to supplement short comings and allow the full free energy landscape of the active matter systems, such as EAP hydrogels, to be utilized. The use of EAP hydrogels in this way is currently unexplored; however, similar exploitations of material morphological computing have been shown with great potential. EAPs unique properties have already been extensively explored as building materials in many biologically inspired robotics,^38^utilizing EAPs morphological behaviors in the production of biomimetic soft body robots from worm-like^39^ to jellyfish^40^ designs and all manner of micro robotics.^41^

Recently, the computational abilities of pneumatic and compliant soft robotic media have been exploited to aid in their own control,^42^^,^^43^ and then expanded through the use of EAPs to improve this “self-control” by constructing physical reservoirs.^44^ The computational advantages of EAPs have been further exploited in applications such as biomedicine and e-textiles,^45^ where in the latter the ability for EAPs to act as both actuator and sensor are combined with morphological computation to allow for unique integration with clothing. Ionic EAPs have been explored as stress sensors utilizing their inherent morphological nature to allow for more versatile and sensitive detection.^46^ EAP hydrogels exist as a type of ionic EAP and exhibit behaviors found in the majority of these technologies such as actuation, sensing, compliance, and computation. However, EAP hydrogels have yet to be fully explored for these applications because of the lack of existing frameworks to support them. The applications mentioned above (biomimetics, micro robotics, e-textiles, and biomedicine) would serve as fruitful applications for EAP hydrogel computation technology.

In summary, computation is not only limited to the most common implementations, a vast field of morphological and embodied computing theories allow computation to be realized in a great number of unlikely places. Active matter systems are one area of these new media that present computational capability through morphological theories. EAP hydrogels are an active matter system showing much potential in this computational application. Utilizing the ubiquitous computational frameworks of automaton, the computation of active matter systems can be exploited as shown in chemical examples.^19^ With more appropriate automaton frameworks, such as probabilistic Moore automaton, can other active matter systems, such as EAP hydrogels, be exploited for computation beyond the currently explored EAPtechnologies. Furthermore, utilizing additional morphological computing structures such as reservoir computing, it is possible to further utilize the potential of such an active matter medium.

This study aims to be a pioneering work into applying the unique mechanics within EAP hydrogels by developing and validating an automaton reservoir framework, designed to harness the EAP gels responses to an electric field. First, using the mechanical responses of the ionic EAP hydrogel under an electric field, we studied the memory mechanics present within its behavior. Second, we applied this memory to develop a probabilistic Moore machine automata structure and evaluated this structure against the hydrogel’s responses to stimuli. This allowed us to analyze the hydrogel’s computational potential as an automata. Third, we expand the automata framework through reservoir computing and thresholding to construct a hybrid automaton-reservoir system capable of beneficial computation.

## Results and discussion

### Memory mechanics through ion migration

Polyacrylamide hydrogel is an ionic, active matter, EAP that has been shown to exhibit large shape changes.^21^ As an ionic EAP, the volume changes are induced through ion migration, followed by osmotic pressure driven water flow. Hydrogen ions act as active agents within the hydrogel, interacting with the polymer networks. Polyacrylamide is a relatively simple EAP hydrogel to synthesise,^47^ allowing for it to be produced in large batches and moulded in many different ways for the required experiment. After polymerization, these hydrogels are inert^48^ making them easy to handle. Most importantly the electric field stimulation only requires small voltages to operate, being stimulated by tens of volts depending on size^21^ whereas many alliterative EAP materials such as dielectric elastomers require in excess of a thousand volts.^49^

With stimulation by an electric field, the ions migrate^50^ as a result of the combined electric field^21^ and ion chemical potential.^51^ As the ions move, they drag water molecules causing changes in water distribution. As the ions accumulate on one side, the gel swells and deforms. Swelling is driven by equilibrium between osmotic pressure^52^ and rubber elasticity^53^ in the polymer network. Temperature and water content within the hydrogel can affect this equilibrium.^21^ Temperature alters the elastic properties and rate of ionic motion.^47^ As the osmotic pressure difference between the polymer networks and ionic solution drives the swelling, changes in the hydrogel’s water content change the degree to which it can swell, as well as altering the mechanical properties of the material.^54^ Because of this, the temperature and water concentration must be controlled; this is detailed in the STAR method section.

Ions entering a location cause an increase in osmotic pressure which, in turn, causes water molecules to enter the location and drive swelling. As a location swells, larger volume changes are required to increase the width by the same amount, meaning larger osmotic pressures and larger quantities of ions. Given a constant migration rate of ions into a local polymer network, the localized rate of swelling gradually decreases creating a hysteresis effect.^22^ Because of this hysteresis effect if a series of stimulations were applied, of consistent length and strength, each consecutive stimulation would cause less localized swelling in the gel than the previous. Because each consecutive stimulation causes less and less volume change, each deformation has influence on further deformations.

Although the gel takes little time to swell with stimulation it takes considerably longer to deswell without stimulation, this is demonstrated in the Figure S1. The difference in time scales allows previous stimulations to affect future stimulation-deformation cycles. Thus, the hysteresis effect is driven by the slow diffusion of counterions in and out of the local polymer networks, this in turn leads to a memory function in the hydrogel’s behavior.^55^ This memory function can be demonstrated by recording the collection of ions via the voltage potential across the gel as electric stimulations are applied, the procedure for which is discussed in the STAR Methods section.

**Figure 1:**
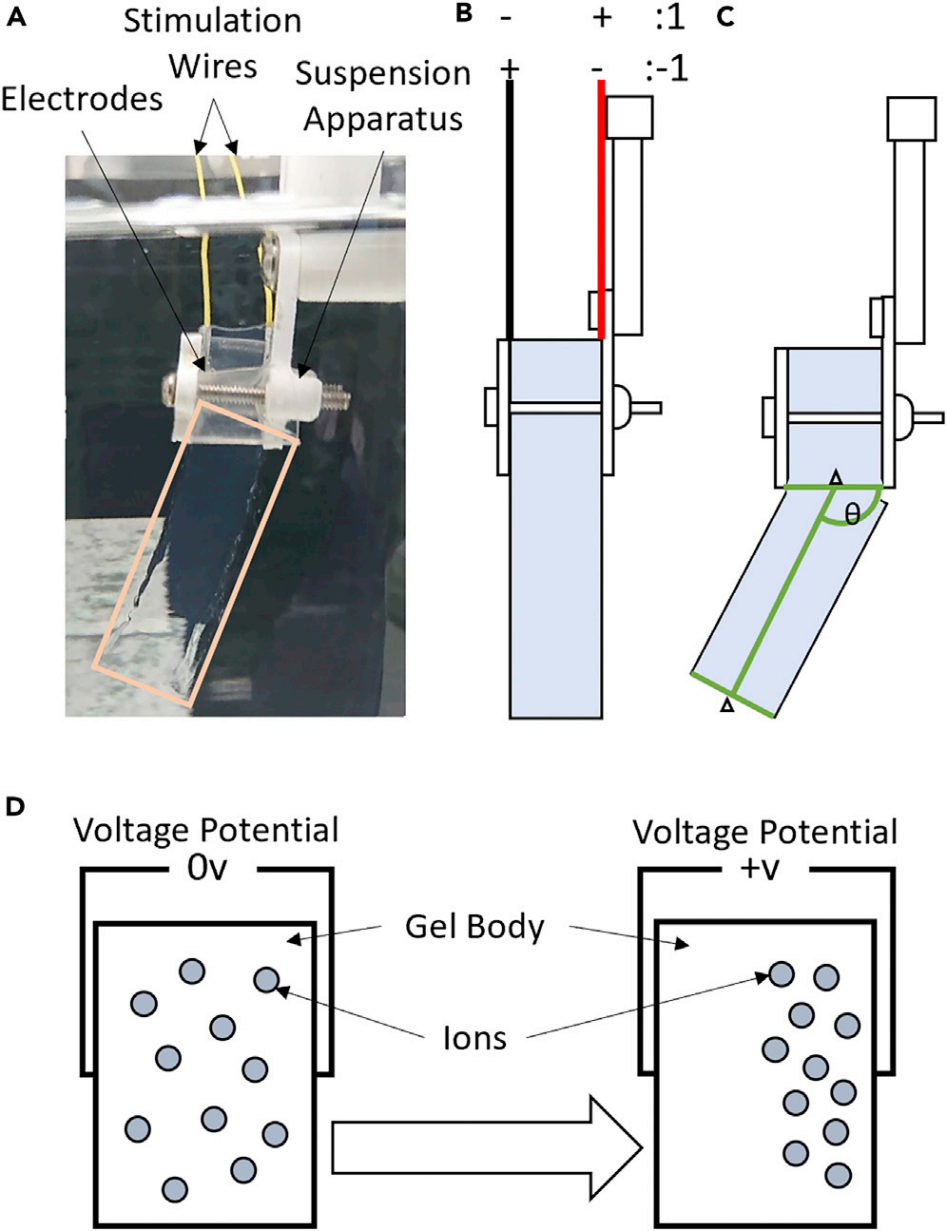
Illustration of experimental EAP hydrogel motion in reaction to electric stimulation as driven by ion migration **(A)** EAP gel suspended within the solution; gel is highlighted by yellow outline. 3D printed apparatus holds the gel on electrodes at set height within the solution, yellow wires provide power to electrodes for stimulation. **(B)** Diagram showing the symbol representation of stimulation polarity, 1 is +31 V and −1 is −31 V. **(C)** Diagram showing how gel bending angle is measured against base of suspension apparatus and gel's mid-point at tip. **(D)** Diagram showing how inhomogeneous distribution of ions causes change in the voltage potential between gel’s surfaces.

**Figure 2:**
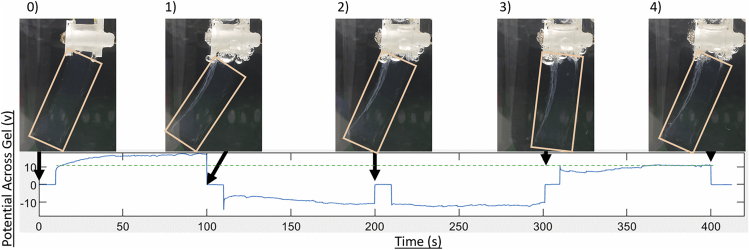
Voltage potential across gel over time Key frames from recorded video illustrate bending at t = 0, t = 100, t = 200, t = 300 and t = 400 (seconds) and labeled 0,1,2,3,4 respectively. A green dashed line is used to compare the voltage potential at end of a stimulation with that at beginning. The sequence applied in the experiment show is −1,1,1,-1.

Voltage potential is directly proportional to the distribution of ions, a concentration of charged ions at one electrode creates a potential much like an ion battery,^56^ demonstrated in Figure 1 section d. Figure 2 shows a sequence of positive and negative stimulation of (−1,1,1,-1) where 1 represents the electric stimulation of +31 V, and −1 the stimulation of −31 V.

For each stimulation, the electric potential (voltage) was recorded against time. The electric potential was measured using the same electrode pairs which applied the electric stimulation (Figure 2). Another stimulation sequence can be found in the Figure S2 (representing a stimulation sequence of −1,-1,-1,1).

In Figure 2 pictures of the gel’s responses at the different time-steps were labeled from 0 to 4. Gel behavior is described corresponding to the following time steps below:

0. At t = 0s in Figure 2, gel has yet to be stimulated and was at default position.
1. At t = 10 S in Figure 2, positive stimulation (31v) was applied for 90s placing anode on right until t = 100 S. Positively charged ions were drawn to negative electrode (right) pulling water molecules with them causing swelling and the gel to bend to the left. There was an immediate rise in voltage potential provided by electric field, as ions gather on right the voltage potential increases further.
2. At t = 110 S in Figure 2, negative stimulation (31 V) was applied for 90 S placing anode on left until t = 200 S. There was an immediate drop of voltage potential shown in the figure at t = 110 S caused by application of the electric field. Ions were currently toward right, so voltage potential starts higher (closer to 0 as shown on graph). Ions were pulled to left electrode causing voltage potential to decrease as they gather causing swelling on the left and bending the gel toward the right.
3. At t = 210 S in Figure 2, positive stimulation (31 V) was applied for 90 S placing anode on right until t = 300 S. Ions continued to move toward left electrode causing further swelling on left, further bending the gel to the right. The hysteresis effect can be seen in this change in voltage potential. The voltage potential first plateaus, then starts rising toward 0, as the minimum limit of voltage potential reduces. The limits of the voltage potential (directly related to the swelling amount via ion migration) reduce over continuous stimulation by the hysteresis effect.
4. At t = 310 S in Figure 2, positive stimulation (31 V) was applied for 90 S placing anode on right until t = 400 S. Ions moved back toward right electrode causing swelling and the gel to bend slightly left, but less, because of the hysteresis effect caused by the change in ion distribution as a result of swelling. Voltage potential raised as ions gather but because of the hysteresis, the level of the potential was less than that achieved by the initial stimulation (shown by the dashed green line).

**Figure 3:**
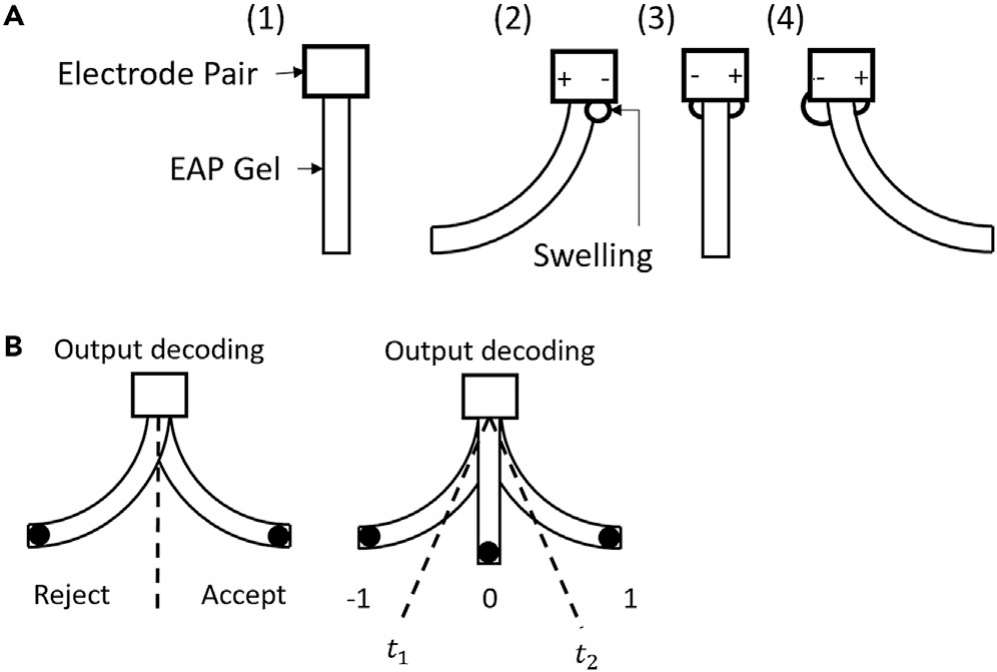
Illustrated affect of compound swelling and encoding of resultant bending angle through threshold application **(A)** Diagrams demonstrating the experimental set-up and effect of compound antagonistic swelling; (1) Experimental set-up with the placement of electrodes on gel, (2) Bending of the gel upon application of electric field, (3) Equalized bending upon electric fields application in the opposite direction, (4) Further bending by continuing application of electric field. **(B)** Application of the thresholds to determine the output series; (left) Acceptance or rejection with one threshold, (right) Symbolic encoding with two thresholds.

The results of Figure 2, described above, show how the electric field induced swelling is subject to the hysteresis through the reduction of the maximum and minimum voltage potentials as a function of consecutive stimulations. The voltage potential necessary to induce the same angle of bending was altered with each stimulation as a function of time, influenced by previous simulations leading to a response tantamount to memory. The influence of sequential swelling can be seen in Figure 3 section A. The same actions can be seen occurring in the Figure S2 (representing an input sequence of −1,-1,-1,1) where the hysteresis moves the voltage potential toward 0 over sequential stimulations (represented by the green dashed line). Now that the memory function has been established through the dynamical responses of the gel, a suitable framework is required to utilize the memory for computation.

### Applying EAP gel to a probabilistic Moore machine automata

One of the most common memory based computational frameworks is the automata. The previous section shows how EAP gels exhibit memory. As a next step, we map electric stimulation and mechanical responses of the gel onto the automata framework to examine the computational ability of the hydrogels. Electric stimulation is considered as the input to the automata system, and the bending angle as the system output. Using this input-output relation, we apply the memory function, attributable to the dynamical responses of the gels, as an automata. Further, in this context, the gel acts as an interpreter, converting one language to another.^57^ As an interpreter the gel converts input stimulations to output postures through the memory function. The memory function of the gel acts as a set of defined instructions. This framework also follows the structure of a transducer automaton.^58^ Because of the highly dynamic nature of the EAP gels, their responses to the electric stimulation have variation in terms of bending angles. A probabilistic automata was used to take account of this variation in behavior and provide an additional dimension in behavior. In probabilistic automata each state transition has a probability of occurring, this means that two identical probabilistic automata would have variations in outputs^26^^,^^27^ mimicking variations between gel instances. We consider that this form of automata can be mapped onto the EAP gel behavior, because the output angles are not purely determined by the input stimulation but also determined by probability as a result of the highly complex dynamics of the hydrogels in an ionic solution.

Automata utilize symbolic representations of inputs and outputs. For example, the output series can represent words in the sentences that are translated by the automata. In automata the input and output sentences can utilize different grammar rules and thus represent different languages,^24^^,^^59^ this is also the case for the hydrogel. The inputs and outputs of the hydrogel require different encoding rules as they represent different energy forms, electrical stimulation and mechanical motion, different encoding rules mean different grammar rules and so different languages.

To fully utilize the gel in an automata framework, we first define these languages in the input and output series of the EAP gels. The gel’s behavior must be broken down into finite states by applying discrete time intervals, defining characters, words, and sentence within the context of gel’s response to a series of electric stimuli as follows; input to the system is voltage applied to the gel as electric stimulation. In this study we used three input states with set magnitude; negative (−31v), neutral (0v), and positive (+31v) simplified into the symbol set of −1,0,1 respectively. The state of the system is defined as the bending angle of the gel under the electric field, and can be mapped, through thresholds, to an output symbol. A demonstration of this mapping is shown in Figure 3 section B, output symbols were set by applying zones over the full movement range. Defining more zones to categorize the bending angles creates an output language with more output symbols, giving higher resolution. However, this higher resolution is at the cost of accuracy because of the probabilistic nature of the hydrogel as an automaton. These thresholds act to define the output symbols, converting an analogue input-output system to a discreet representation adapted to an automaton framework. The thresholding algorithm was used to generate the grammatical structure of the output symbol series. For this study, three thresholding zones were used: left, center, right as −1,0,1 respectively to generate the output symbols as shown in Figure 3 section B in the right diagram.

To utilize these automaton languages a suitable automaton framework is required that is probabilistic and takes inputs for state transitions. Moore machine automata are a restricted type of finite-state transducer, whose output are determined only by its current state.^30^ This behavior closely matches the EAP gel’s automaton behavior, given the constructed language definitions. Moore machine automata are, however, deterministic for a given initial state and input pair, a deterministic finite automaton (DFA) has exactly one next state.

The dynamical responses of the EAP gels are non-deterministic or probabilistic; however, a probability component can be introduced to the Moore machine definition to create a Probabilistic Moore Automata (PMA). The PMA can be defined using an adapted Moore machine definition that includes a probability component P mapping probabilities to the transition function. This is shown in Equation 1 as a 7-tuple system.

(1) $$\begin{array}{c} A=\left(Q,\Sigma ,\Gamma ,\delta ,P,\omega ,\iota \text{,}\right)\;Where\text{:}•States\;Q=\left\{S_{01},S_{11},S_{12},\ldots ,S_{NI}\right\},I=2^{N}\\ \;where\;S_{i,j}\in Q\;for\\ \left\{i\in Z\;|\;0\leq i\leq N\right\}\;and\;\{j\in Z\;|\;1\leq j\leq I\},\\ and\;N\;is\;the\;sentence\;length\;N\in Z^{\geq 0}•Input\;alphabet\;\Sigma =\left\{-1,0,1\right\}•Output\;alphabet\;\Gamma =\left\{-1,0,1\right\}•Transition\;function\;\delta :Q\times \Sigma \to Q•Transition\;probabilities\;P:\delta \to R^{+}•Output\;function\;\omega :S\to \Gamma •Initial\;state\;\iota =S_{0,1} \end{array}$$

For every symbol in the input sentence, the active matter moves to the appropriate state based on the probability of the transition, and gives the output associated with that state. The output function ω defines the encoding that maps from state to output via thresholding, translating gel bending angle to output symbol.

To evaluate the application of the probabilistic automaton on the EAP hydrogel, the gel is stimulated using the defined input sentence, and the responses compiled, details on the experiment are found in the STAR Methods section. In this experiment N was set to be 3, as stimulation beyond that point led to no significantly measurable response from the EAP gel. Thus, the input and output sequences were defined as the 3 element vectors $\left\{I\left(l\right):I\left(l\right)=\left(I_{1},I_{2},I_{3}\right),I_{i}\in \Sigma \right\}$ and $\left\{O\left(k\right):O\left(k\right)=\left(O_{1},O_{2},O_{3}\right),O_{i}\in \Gamma \right\}$ respectively, where i is the $i^{th}$ symbol in the vector. l&k are labels for the input and output state respectively as a result of the sequence vector, given by Equations 2 and 3.

In Equation 2, l is the integer labeling the state of the input vector I and $I_{i}$ is the $i^{th}$ symbol in I, for example, I(5)=[1,−1,1] and I(2)=[−1,1,−1]. In Equation 3, k is the integer labeling the state of the output vector O and $O_{i}$ is the $i^{th}$ symbol in O, for example O(5)=[−1,1,0] and O(21)=[1,0,−1]. The experiments seek to create a generalized framework to overcome inconsistencies between gels, and so, each series of 8 input permutations is repeated with a different batch of gels.

(Equation 2) $$l=\sum_{i=1}^{N}2^{i-1}\left(I_{i}+1\right)/2$$

(Equation 3) $$k=\sum_{i=1}^{N}3^{i-1}\left(O_{i}+1\right)$$

**Figure 4:**
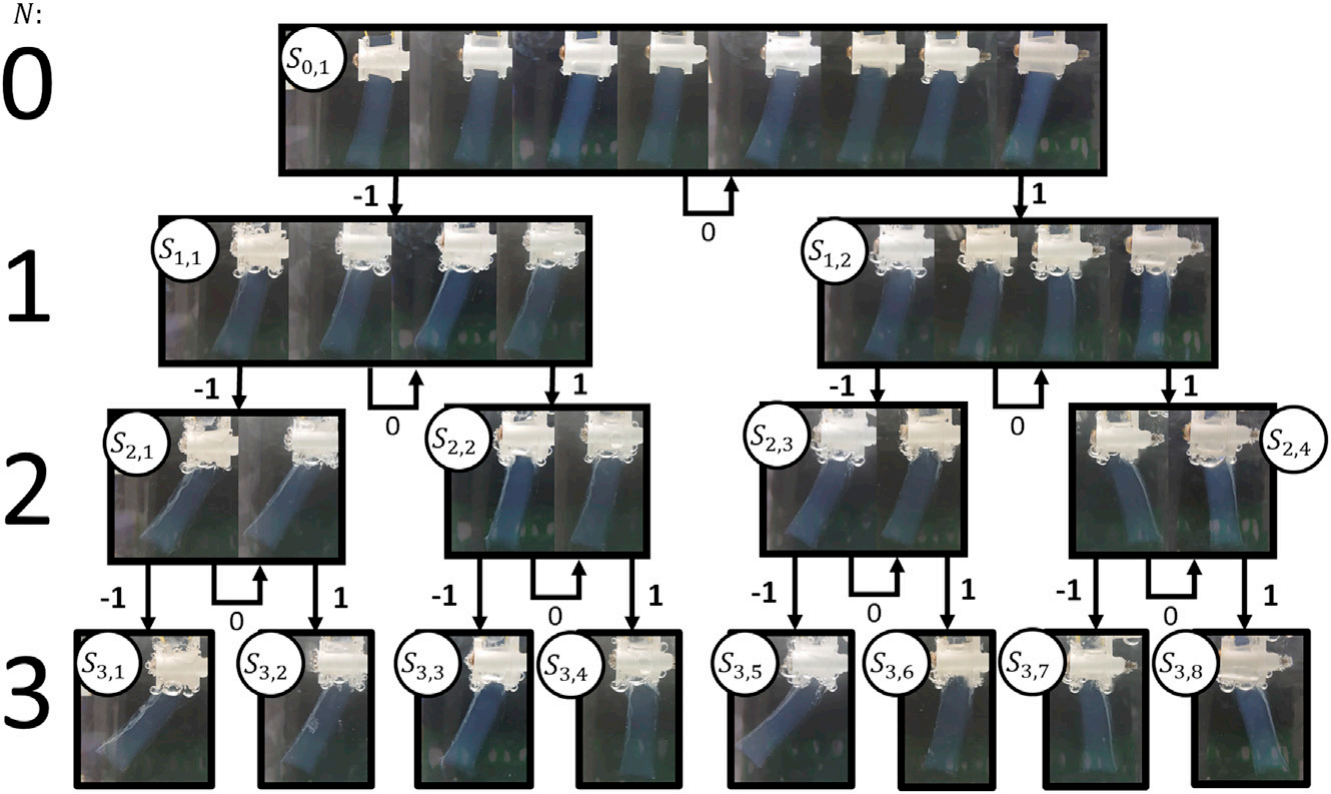
Automata directed graph for a 3-symbol system (N = 3). 8 gels were run to explore each sequence and branch of the directed graph At each stage of the sequence the gels are shown side by side in the same order for each layer. Each $S_{i,j}$ node represents a state of the machine where i denotes the layer (or symbol in the case of the input/output sequence) and j denotes the path that led to that state for that layer, with $S_{0,1}$ as the initial state, also defined in Equation 1. Each node has 3 input paths; −1 and 1 lead to subsequent states, 0 representing no input leads back to the same state.

Each series of 8 different input sequences creates its own state tree of gel postures which are compared with each other to evaluate consistency and repeatability. One such state tree is shown in Figure 4 and depicts the change in gel posture with different input sequences as a directed automata graph.

To effectively judge consistency of the dynamical responses between gel samples, some post-processing is needed to filter noise from unavoidable inconsistencies between experiments e.g. positioning of gel, gel surface texture, slight synthesis differences between batches etc. The procedure is designed to minimize inconsistencies. To filter noise in the current setup, a gain α and offset $\theta_{0}$ parameter are used to reduce the effect of differences in surface texture that cause initial bending in the gel at t = 0.^60^ The use of these parameters also reduces the effect of differences in synthesis between batches because of differences in chemical ratio changing in elasticity,^61^ respectively. The specific use of this filtering can be found in the supplementary information in Methods S1″Post-Processing Experimental Results to Reduce Variance”, the reduction in variance via the post-processing can be seen in the supplementary information in Table S1. From this filtering the variance^62^ in output angles for each input sequence was reduced. Thresholds are used to categorize the continuous output into a symbol list to be used in an automaton context, as represented by the output function ω in equation set 1 and defined by Equation 4, where O is the output symbol, Θ is the post-processed output angle, $t_{1}$ and $t_{2}$ are thresholds 1 and 2 respectively. A smaller symbol list allows for reduced variance, but also, reduces computational ability as fewer symbols can be used to represent computed results. Likewise larger symbol lists allow for more complex computation but are more sensitive to gel composition.^63^ There needs to be a balance between computation and predictability.

(4) $$O=\omega \left(\Theta ,t_{1},t_{2}\right)=\left\{\begin{array}{cc} -1 & \Theta <t_{1}\\ 0 & t_{1}\leq \Theta \leq t_{2}\\ 1 & \Theta >t_{2} \end{array}\right\}$$

To apply the collected data to the probabilistic automaton structure, $t_{1}$ and $t_{2}$ must be set in Equation 4. Selection of output thresholds alter the response of the gel as an automaton. To assess the automata’s ability under different values of $t_{1}$and $t_{2}$, the following criteria was used for optimization:

• Maximize predictability: Achieved by maximizing the probability that each I vector will consistently result in the same O vector between activations of the probabilistic Moore machine.
• Maximize computational range: Achieved by realising equal distribution of output symbols in the O vectors generated by the probabilistic Moore machine.
• Maximize computational versatility: Achieved by maximizing the number of unique O vectors given by the probabilistic Moore machine across all I vectors.

Through the optimization of these criteria the thresholds were found to be −7.8 and 2.7 for threshold 1 and 2, respectively. A more detailed definition of the optimization function approach and results can be found in the supplementary information in Methods S2 ″Optimization of Output Encoding Thresholds to Maximize Automaton Response”, the results of the optimization of these criteria can be seen in the Figure S3. From these thresholds a bias can be observed toward one direction as the negative threshold is much larger than the positive. This is likely because of the orientation of the gel in the molding process, the bottom has contact with the mold surface whereas the top is open leading to a difference in surface texture. However, as all gels are made consistently this shouldn’t affect the validity of the results.

**Figure 5:**
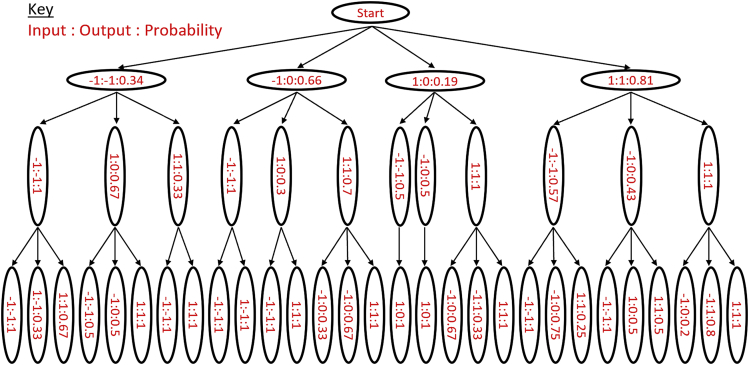
The directed graph of the probabilistic automaton Using thresholds −7.8 & 2.7 for thresholds 1 $\left(t_{1}\right)$& 2 $\left(t_{2}\right)$ respectively (as found through threshold optimization detailed in the supplemental information Methods S2 ″Optimization of Output Encoding Thresholds to Maximize Automaton Response” and Figure S3). The starting state that the gel inhabits is labeled ”Start”, each following node depicts a possible state which is labeled in red with the following format (Input, the input symbol that lead to this state from the previous state): (Output, the output symbol given from entering this state): (Probability, the probability that this state would be entered given the input symbol).

The optimized thresholds were used to generate a transition tree from the experimental data and generate the probability transitions as shown in Figure 5. The probabilistic directed graph is of Figure 5, showing the probability that each transition path will be taken given a certain input symbol and the function of the hydrogel as a non-deterministic automata. This resultant probabilistic automaton shows a promising complexity in the automata’s output response and rule structure, with threshold selection able to tune the ability of the automaton.

When designing automata, the complexity of the program (rule set) is limited by the range of possible responses. With a probabilistic state system, the possible responses are far more versatile. There is, however, a trade-off, the automaton has a much wider range of computational capacity (able to output more complex sequences) at the cost of accuracy giving less determinable results. This means that the use of such a computational resource needs to be fit for purpose regarding application and computational ability. In the next section, to broaden the computational ability, we expand the framework to better embody the mechanisms of the gel as a computational reservoir.

### Probabilistic Moore automata as a computational reservoir

The PMA is rich in dynamics, allowing information storage through memory of the EAP hydrogel. Each state of the directed tree acts as a non-linear node encompassing the behavior of the non-linear units (active ions) as shown in Figure 5. This network of nodes behaves similarly to a neural network and, more specifically a reservoir. Reservoir Computing is a framework that maps input signals into higher dimensional computational spaces through the dynamics of a fixed, non-linear system called a reservoir.^34^ Reservoir computing is a derivation of recurrent neural networks and as such implements the ability to learn. In reservoir computing learning takes place through the optimization of output encoding.^35^ The encoding functions are altered until the result best embodies the function desired for the intended application.

Reservoir computers allows for highly dynamic analog systems to be used in computation, allowing them to perform computations that would otherwise be slower or less accurate on digital computers.^35^ These computers are, however, less versatile than current digital systems as the selection of a reservoir is dependent on the intended application. For example, the ripples in a vibrating water source have been used to encode image data more efficiently than conventional digital computers.^36^ A reservoir computing layout consists of 3 main components; excitation layer, reservoir, and readout layer,^34^ these components are described in Table 1.

**Table 1:** Description of reservoir computing components with application within the EAP gel network

Component	Description	Application to Gel
Excitation layer	Input to the reservoir, converting from defined input symbols to stimulations in the reservoir network.	Inputs are defined as −1,1 and are applied via electrical stimulation across the width of the gel. This is the same system as the excitation layer used in the Moore Machine framework.
Reservoir	Fixed, non-linear system that is used to map inputs to higher dimensional space.	EAP hydrogel suspended in the ionic solution via surface electrodes, stored as a virtual database through the PMA.
Readout layer	Output from the reservoir. Converts from the reservoir network to defined output symbols. This layer is tuned to train the reservoir computing network.	Bending angle of the gel as viewed from the side perpendicular to the electric field, binarized (thresholded) into the defined output symbols of −1,0,1. Tuning is performed through the optimization of thresholds.

A reservoir in this context must be made up of individual, non-linear units, and capable of storing information^64^ much like the PMA has already presented. In the field of physical computing, reservoirs produce multiple outputs to provide a rich non-linear in interaction with the surrounding environment. For example, down the length of an octopus arm^10^^,^^65^ or the location of ripples in a vibrating water source.^36^^,^^66^

In contrast, in the current implementation of the EAP hydrogel framework, there is only one physical output via the bending angle of a single EAP gel. However, through using the PMA, additional outputs are given over temporal space. Each symbol of the output sequence of the PMA constructed in the previous section represents an instance in the EAP hydrogels motion. Thus, using the temporal responses of the hydrogels, we can utilize multiple outputs over the duration of the motion path. Figure 5 shows this temporal sampling as each layer of the tree represents a different step of time in the automaton’s operation. Figure 5 also shows each path has an associated transition probability that increases the dimensionality of the automaton. This means the relatively simple EAP system can be used as a reservoir via the generated PMA, providing the required multiple outputs via temporal sampling and higher dimensions via the probability element. The similarities between the PMA and reservoir computing frameworks allow for techniques of reservoir computing to be used to expand the computational ability of the PMA by using it as a reservoir. To apply this reservoir framework to the EAP gel based PMA, the reservoir computing components must be defined in terms of the EAP gel system, these comparisons are shown in Table 1.

The main concept of reservoir computing is to utilize the non-linear behavior of a physical system to do computations that would require an approximated mathematical model to calculate^35^ via conventional purely digital/binary computation systems, such as microcontrollers or personal computers. For the PMA to have value as a reservoir, its behavior must embody computations that would otherwise be less effective when modeled via conventional digital computational means. The mechanics of the EAP hydrogels showed continuous probabilistic responses upon electrical stimulation, which were discretized to fit the automaton framework. Although most examples of reservoirs in morphological computing are physical, like the gel, they can also be represented virtually.^64^ Virtual reservoirs can take the form of modeled systems, such as artificial neural networks, or as a database of responses from physical reservoirs. The discretized probabilistic automata transitions from the previous section, as shown in Figure 5, have potential as a virtual database reservoir that can be used to validate the EAP hydrogel PMA as a reservoir computer. The use of a virtual database reservoir allows for faster analysis of the complexity of a PMA reservoir based on EAP hydrogel.

To utilize the PMA network, as shown in Figure 5, as a database reservoir the input and output sequences need to be simplified to allow for easier comparison to conventional digital computation. For the purpose of simplification, the input and output sequences are represented by the state labels l and k described in Equations 2 and 3 for input and output sequences, respectively. The PMA takes a sequence of inputs (I) and maps them to a sequence of outputs (O) utilizing a network of weighted transitions. Using the state labels l and k the PMA becomes a non-linear integer mapping function based on the PMA’s probability distribution, derived from the recorded responses of the EAP hydrogel. The non-linear function of the integer mapping becomes the database representation of the EAP hydrogel reservoir, where the output layer is adjusted through tuning the thresholds $t_{1}$ and $t_{2}$ in the output function ω, as defined in Equation 4.

**Figure 6:**
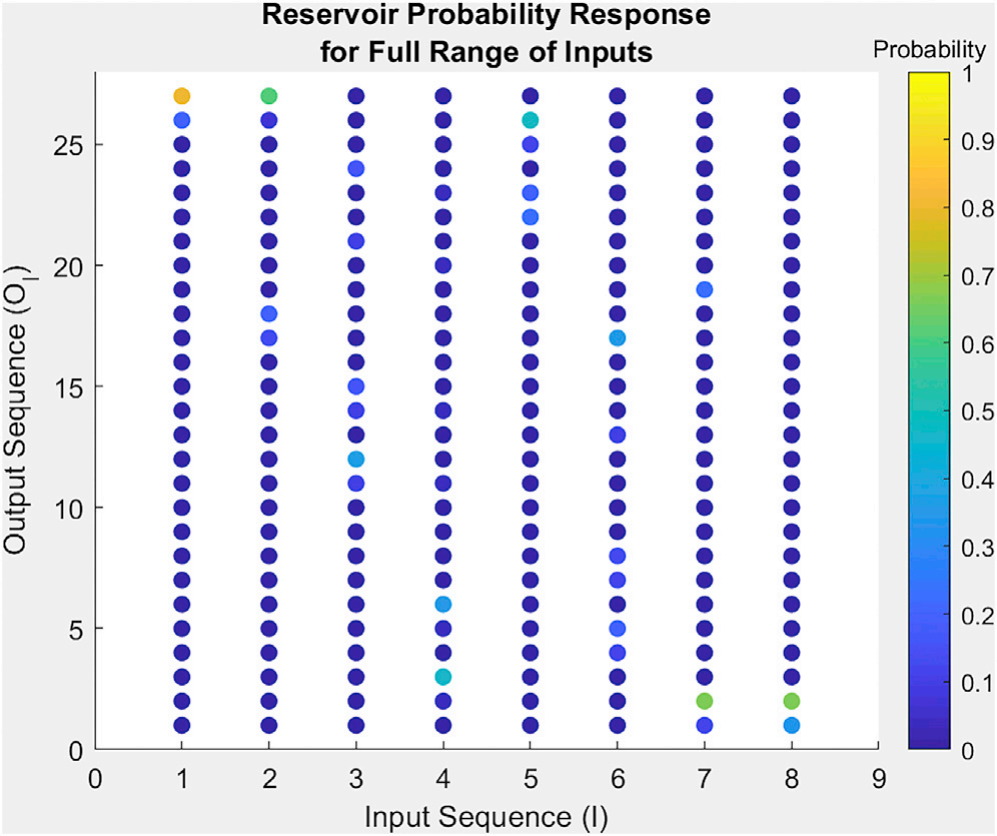
Probability of each output integer given an input integer using thresholds −7.8 & 2.7 for thresholds 1 & 2, respectively The probability is shown as a color representing a number between 0 and 1.

In the function of the PMA, the thresholds served to binarize the analog output of the gel to fit a sequential symbol language. However, in the context of a reservoir tuning the output layer, through the adjustment of $t_{1}$and $t_{2}$, tunes the probabilistic mapping between input and output states. By tuning this probabilistic mapping, the function of the reservoir can be controlled to serve a purpose. In more general terms, the reservoir as a whole embodies a large energy landscape of probability maps, but through the adjustment of the output layer can be tuned to a specific probability mapping that can be used within a computational application. An example of a single probabilistic mapping condensed from the reservoir via the thresholds $t_{1}=-7.8$ & $t_{2}=2.7$ is represented in Figure 6, where the input and output sequences are denoted by their state labels. Shown in Figure 6, for each input state, each output state has a probability of occurrence. For each input state, there is a single leading output state that has a much higher probability of occurrence than the other output states. With a larger dataset, it is likely that these outcomes converge further, and this leading output state will become more pronounced as the minor differences in gel synthesis and experimental procedure become less impactful on the final results.

This framework provides a virtual reservoir representation of the complex continuous function of the EAP hydrogels computation. By tuning the thresholds, the reservoir provides a mapping function of 8 input states to 27 possible output states. However, for the EAP reservoir to be useful as a computational medium it must be capable of performing certain computational tasks more efficiently than purely digital/binary based hardware alternatives. The EAP reservoir is structurally simple, comprising of only 3 layers in the automaton, although the analog nature allows infinite combinations of input and output sequences when continuous values are considered. For example, by changing the input/output language through the adjustment of the stimulation voltages or output bending angle thresholds. A digital computer would never be capable of exactly representing an analog system, but in practice an approximation of analog results is often sufficient. Any commonly used digital computing system can represent a function through models stored in memory. Given a digital system with enough memory, any analog system could be approximated sufficiently to be used in a given task. To compare the EAP reservoir with digital systems the question is, how large would the memory of a digital system need to be to accurately represent the EAP reservoir. A comprehensive answer to this comparison depends on the application, but by comparing the mapping functions generated by the EAP reservoir with a model commonly used in digital computation systems the difference in memory usage can be visualized.

One of the simplest forms of model that is used within digital computation systems is the polynomial. Because of polynomial’s simplicity they can be represented on any form of digital computer hardware, from simple microcontrollers to complex cluster computers. All digital systems use memory to store functions, in the case of the polynomial it’s the coefficients that require memory space to be stored. The mapping function generated by tuning the thresholds of the reservoir can be compared to the polynomial representation of the same function, comparing the memory usage and accuracy of the representation in each case. In the physical reservoir the mapping functions are continuous like the hydrogel but have been discretized for the purpose of simplifying the initial framework. This discretization also allows for virtual reservoir storage and more direct comparison to digital computation. If the polynomial requires as much memory as used by the mapping function, generated from the virtual representation of the reservoir, then for that function the reservoir is more efficient in memory usage than the digital polynomial representation. Which is to say, the hydrogel computation is more efficient than the same computation achieved utilizing digital computation methods. The memory used in the representation can be correlated with the number of coefficients required to represent the model. As there are only 8 possible inputs, using 8 coefficients (7th degree polynomial) would essentially create a lookup table and be no more computationally advantageous than the virtually stored EAP reservoir. For this reason polynomials with degrees from 0 to 6 will be tested and their performances assessed.

There are many different modeling methods with some far more optimized; however different methods require different hardware to run. Polynomials, as one of the simplest modeling techniques, can be implemented on any hardware. This means that using polynomials as the comparison allows the assessment of the hydrogel reservoir to be hardware agnostic, allowing for a more generalized conclusion for this initial investigation. In addition, the EAP reservoir already represents the free energy landscape of the physical system which, in this case, is used to generate the computation, selected by tuning the thresholds. The hydrogel does not have to generate this energy landscape as it is inherent to its structure, whereas in the digital system a computer must generate the landscape representation through the calculation of the polynomial approximations. Because the computational resources used to generate the polynomial approximations of the EAP reservoir have no counterpart in the hydrogel’s computation, they will be ignored when comparing efficiency.

To assess the entire free energy landscape of the EAP reservoir, each combination of thresholds needs to be converted to a reservoir instance and represented digitally for evaluation. To accomplish this, a database of PMA reservoir instances is made containing the probability distribution (as shown in Figure 6) of the reservoir at every threshold combination. This uses the same threshold range as used in the threshold optimization earlier in the study, using the minimum to maximum angle reached as the bounds (−31.3 and 29.3) with a step of 0.5°. The thresholds are applied with Equation 4 where $t_{1}$ and $t_{2}$ are the thresholds constants for that iteration of the automata.

(Equation 5) $${\bar{k}}_{l}=\left\lceil \left(\sum_{k=0}^{3^{N}}P\left(k,l\right)k\right)+0.5\right\rceil$$

(Equation 6) $$\bar{k}=\left({\bar{k}}_{0},{\bar{k}}_{1},\ldots ,{\bar{k}}_{l},\ldots ,{\bar{k}}_{\left(2^{N}-2\right)},{\bar{k}}_{\left(2^{N}-1\right)}\right)$$

To represent each EAP reservoir instance in the database as a series of polynomials, the probabilistic component will need to converted to analog outputs. To this aim, we obtain the average output label for each input sequence using Equation 5, based on the assumption the probability transitions converge as predicted from the interpretation of Figure 6. Equation 5 defines how the probability distribution of output labels for a given input label are averaged to a single output label to create a one-to-one mapping that the polynomial can attempt to model as a continuous function. ${\bar{k}}_{l}$ is the average output state label representation k for the input sequence labeled l as found from Equation 2, the average output labels are stored as a vector $\bar{k}$ defined in Equation 6 where $\left\{{\bar{k}}_{l}\in Z|0\leq {\bar{k}}_{l}\leq 26\right\}$. As with the PMAs definition before N=3. In Equation 5P(k,l) gives the probability that the output state k is given when the PMA receives the input sequence l, as derived from the probability distribution of PMA outputs for a given set of thresholds.

(Equation 7) $$f\left(l\right)=\sum_{p=0}^{r}a_{p}l^{p}$$

(Equation 8) k=⌈f(l)+0.5⌉

(Equation 9) $$\underset{\left(a_{0},\ldots ,a_{p},\ldots ,a_{r}\right)}{\text{argmin}}\sum_{l=0}^{2^{N}-1}|f\left(l\right)-{\bar{k}}_{l}{|}^{2}\text{,}$$

$$subject\;to:\;a_{p}\in R,a_{p}\in \left[-100,100\right]$$

With the EAP reservoir instances converted to a series of analog mapping functions, they can now be represented via polynomials. The generalized polynomial used to represent the continuous EAP reservoir mapping functions is defined in Equation 7. The polynomial is fitted to the averaged reservoir mapping using the minimization Equation 9 for a given degree r. f(l) is the input to output transition function that maps input values I to output values O, the result of f is rounded to the closet integer (to correspond with the integer representation of output sequences) as shown in Equation 8. pis the power, $a_{p}$ is the coefficient associated with the power p and r is the degree of the polynomial. The number of coefficients used to represent a polynomial function is representative of the memory required to store that function. So, by fitting polynomials, with various numbers of coefficients, to the averaged continuous EAP reservoir mapping, and analyzing how well each degree of polynomial fit the actual EAP reservoir mapping, the effectiveness of representing the EAP reservoir digitally can be assessed. Fitting was performed using the polyfit function in Matlab^67^Polyfit uses least-squares minimization via the discriminant of the Vandermonde matrix to find polynomial coefficients of the specified degree, that most closely represents a given dataset. For each fitted polynomial, the norm of residuals for the error of ${\bar{k}}_{l}-k$ is calculated to represent the accuracy of the fitting for the polynomial f given the inputs I and average reservoir outputs ${\bar{O}}_{I}$. Each averaged reservoir instance in the database is then fitted to a polynomial using degrees from 0 to 6 to visualise how effective digital systems can represent the free energy landscape of the PMA reservoir.

**Figure 7:**
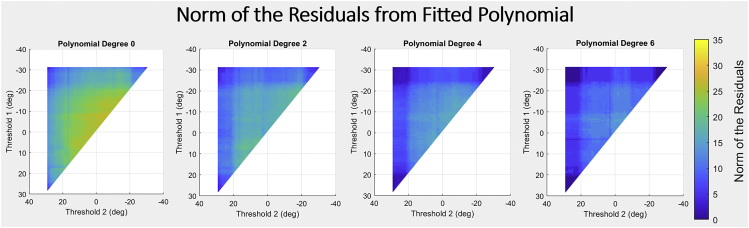
Heatmap for the norm of residuals error of fitted polynomials, against the threshold values used in the output layer of the PMA reservoir instance that the polynomial is fitted to Each pair of threshold values is used to generate a PMA reservoir by being applied to the output layer and a polynomial is fitted to the mapping function of these reservoirs. These graphs show the results of polynomial fitting for polynomial degrees 0, 2, 4, and 6 with graphs 1, 3, and 5 found in the supplementary information figure S4. The color bar shows the relative norm of residual values.

Using this validation method, the graphs in Figure 7 were generated using the degrees 0, 2, 4 and 6 (graphs for 1,3 and 5 found in the Figure S4). As described earlier, each pair of threshold values is used to generate a PMA reservoir instance via the output layer, and a polynomial is fitted to the resultant mapping function of these reservoirs. These graphs show a norm of residuals heatmap for the fitted polynomials against the threshold values in the output layer of the PMA reservoir.

More coefficients allow the polynomial to fit the PMA reservoir’s mapping function better, which is to be expected. The bounds of the tested thresholds (−31.3 and 29.3 for $t_{1}$ and $t_{2}$ respectively) are always the points of lowest error. This is expected as these thresholds represent the extremes of the reservoir. As −31.3 and 29.3 are the maximum and minimum angles achieved by the gel, if the thresholds were set to these values all output angles would be in a single segment of the thresholded ranges (as shown in Figure 3 section B), meaning all input sequences would result in the same output sequence. The ‘texture’ of the heat maps in Figure 7 have consistent patterns of vertical and horizontal lines. These lines are a good representation of changing sensitivity in the reservoir’s response to threshold selection. As the thresholds move closer to recorded output angles of the gel, the impact of the change in threshold value on the reservoir’s structure becomes more significant. From the graphs in Figure 7, the fitted polynomials required a significant number of coefficients to be close in ability, with pronounced error present until a polynomial of 4th degree was used. As the number of coefficients approaches the number of possible input sequences, the polynomial becomes a direct mapping and offers no computational efficiency over the reservoir. This observation gives insight into the kind of hardware memory required to represent the EAP reservoir based on the number of coefficients used.

The reservoir mapping function being fitted is a sampled average approximation of the true EAP hydrogel function, reducing the probability component, and using only a 3-symbol input sequence I with a 2-symbol language. This means that given the simplest form of the EAP reservoir, where the probability function converges causing a simplification of the probability component, the reservoir function would still not be able to be represented by the digital computational method without significant error. From this, it is likely that a purely digital representation of the EAP reservoir in computer hardware, such as with a computer modeling method similar to polynomial representation, would not be able to efficiently replicate the high complexity and rich dynamics of the EAP hydrogel reservoir. This ineffectiveness would be even more pronounced when input and output languages of higher complexity and larger vocabulary are used to encode the responses of the EAP gel.

Utilizing the reservoir in an application provides an interesting problem, as the task the reservoir’s calculation contributes to needs to be possible within the computational landscape of the PMA reservoir. Given a system where events lead to outcomes with certain probabilities of occurrence, such a system can be simplified into a PMA structure. It is unlikely that the probabilities will match that of the EAP hydrogel PMA, but this is where using the PMA as a reservoir allows for it to contribute to computation. The EAP hydrogel PMA provides an energy landscape that can be tuned to a given application by changing the interpretation of the response. Altering the output language definition allows this high dimensional space to be sampled appropriately for the purpose.

In summary, the sequence of stimulations can be encoded into input sequences, then the output language can be continually altered until the same probability response of the replicated system is observed. Once the input language is defined, this refinement of the output language can be performed virtually. The complexity of the output language does not matter as long as, through its definition, the desired system probabilities are present within the resultant mapping function. In this way the full range of the reservoir may not be used, but the computation it accomplishes can be used to represent/predict the behavior of the replicated system.

### Conclusions

This study assessed the computational ability of EAP hydrogels, using polyacrylamide EAP hydrogel to explore the embodied cognition, and how a complex soft body can compute. A framework was designed, using a Probabilistic Moore Automaton (PMA), by constructing a sentence structure for the input stimuli and output response of the gel. Experimental data was collected to generate a PMA, stored as a database of EAP responses. The automaton EAP gel system was shown to be capable of computational tasks, utilizing a form of memory in the gel’s complex dynamics. Through the PMA structure, unique output sequences were generated from the input sequences.

The theory of computation was then expanded to allow for more complex calculation, combining the PMA framework with reservoir computing to form a hybrid system. Reservoir Computing allows the network of active agents within EAP gels to embody a form of parallel processing. The PMA provides a rich free energy landscape that can be tuned to applications through the adjustment of the input and output language definitions. Through analysis of this energy landscape, given the input and output languages defined in the PMA, the reservoir was shown to be capable of functions that would prove difficult to model via traditional digital computational alternatives with the same analog efficiency. With more complex input-output languages the EAP hydrogel PMA would become even more efficient when compared to purely digital representations, providing an opportunity to calculate effectively with the EAP hydrogel PMA reservoir.

### Future works

As the reservoir is constructed using the PMA, it is best suited to applications commonly used with probabilistic automata, such as statistical^29^ or behavior prediction.^68^^,^^69^ Given the mechanisms behind the EAP hydrogel’s computation, it could also be applied to systems that themselves involve memory or consist of many active agents such as for example crowds of people.^70^ There are many interesting possible applications for our approach to utilizing EAP hydrogels for computation. Although the further experimentation required falls outside the scope of this study, the possibilities are worth discussion for future work.

Reservoirs have also successfully been used in image analysis applications for dataencoding,^36^ as a demonstrated reservoir computing application EAP hydrogels could also show much promise in this area. Furthermore, because of the reservoir’s probabilistic nature, there is the possibility for prediction^71^ or recognition,^72^ such as in natural language translation.^73^ These applications would require an image be provided to the reservoir as an input. Our current reservoir implements only 3 inputs so for any significant image more would be required. The reservoirs complexity grows exponentially with additional inputs, but utilizing encoding the number of required inputs could be reduced. For example, compression techniques could be used, regions or kernels could be applied to encode the data via subsampling into fewer inputs, or many fewer complex reservoirs applied in parallel. Similar encoding techniques are used in many neural network image analysis applications.^74^^,^^75^^,^^76^ The outputs would also then need to be processed to decide how the output of the reservoir represents useful information. Through tuning the reservoir’s output layer certain patterns can be highlighted, but the output must be interpreted to usefully extract data. These applications raise many questions regarding the best way to implement the computational ability of the EAP reservoir, each method must be carefully considered for a given application. This study provides a strong foundation and evidence of the potential of EAP gel’s application in embodied computation and future work.

### Limitations of the study

In this study certain avenues were not explored such as: variation in voltages to expand the input language through representation of additional input symbols, additional thresholds to expand the output language through representation of additional output symbols, different arrangements of electrodes, and different EAP gel shapes. All these variations in the setup would add further dimensions to the reaction of the EAP as a computational medium and potentially expand the computational capability as the energy landscape expands. Furthermore, possible applications were discussed, but the additional work required to formatively explore them would not fit within the bounds of this study.

Nomenclature
  α Gain parameter α∈R
  $\bar{\theta_{I}}$ Mean angle for input sequence $I\;\bar{\theta_{I}}\in R$
  ${\bar{k}}_{l}$ Mean output state label given by the input sequence labeled $l\;\left\{{\bar{k}}_{l}\in Z|0\leq {\bar{k}}_{l}\leq 26\right\}$
  $\Theta_{i}$ Processed angle $\Theta_{i}\in R$
  $\Theta_{\max}$ Maximum output angle present in gel in degrees
  $\Theta_{\min}$ Minimum output angle present in gel in degrees
  I Input Sequence $I\left(l\right)=\left(I_{1},I_{2},I_{3}\right)$
  $I_{i}$ Input symbol $\left\{I_{i}\in \Sigma \right\}$
  $O_{I}$ Output sequence $O\left(k\right)=\left(O_{1},O_{2},O_{3}\right)$
  $O_{i}$ output symbol $\left\{O_{i}\in \Gamma \right\}$
  δ Transition relation
  Γ Output alphabet Γ={−1,0,1}
  ι Initial state ι∈Q
  ω Output function
  Σ Input alphabet Σ={−1,0,1}
  $\theta_{i}$ Angle after symbol $i\;\theta_{i}\in R$
  A Probabilistic Moore Automata A=(Q,Σ,Γ,δ,P,ω,ι,)
  a Gain minimization parameter a∈R
  $a_{p}$ Coefficient associated with the power $p\;a_{p}\in R$
  i Symbol $\left\{i\in Z^{\geq 0},i\leq N\right\}$
  k State label representation of O{k∈Z∣0≤k≤26}
  l State label representation of I{l∈Z∣0≤l≤7}
  N Sentence length $N\in Z^{\geq 0}$
  p Power of the coefficient $\left\{p\in Z^{\geq 0},p\leq r\right\}$
  Q Set of Probabilistic Moore Automata States
  R Norm of Residuals R∈R
  r Degree of the polynomial $r\in Z^{\geq 0}$
  $S_{i,j}$ State $S_{i,j}\in Q$

## STAR★Methods

### Key resources table

**Table:** 

REAGENT or RESOURCE	SOURCE	IDENTIFIER
**Chemicals, peptides, and recombinant proteins**		

Acrylamide	Sigma-Aldrich	Cat# A8887-100G
N,N′-Methylenebisacrylamide	Sigma-Aldrich	Cat# M7279-25G
Ammonium Persulfate	Sigma-Aldrich	Cat# A3678-25G
N,N,N′,N′-Tetramethylethylenediamine (TEMED)	Sigma-Aldrich	Cat# T9281-50ML
Sodium Chloride	Sigma-Aldrich	Cat# S7653-250G

**Deposited data**		

Datasets S1 and S2	Zenodo	https://doi.org/10.5281/zenodo.7274655

**Software and algorithms**		

MATLABR2021a	Mathworks	https://uk.mathworks.com/products/matlab.html
MATLAB code developed for this research	Zenodo	https://doi.org/10.5281/zenodo.7274655

**Other**		

Single Output DC Bench Power Supply 0–30V/0-3A -	CPC Farnel	Cat# IN06822
Current/Voltage/Power Monitor Integrated Circuit	Texas Instruments	Cat# INA219

### Resource availability

#### Lead contact

Further information and requests for resources should be directed to and will be fulfilled by the lead contact, Dr. Yoshikatsu Hayashi (y.hayashi@reading.ac.uk).

#### Materials availability

This study did not generate new unique reagents.

### Method details

#### Memory mechanics through ion migration and voltage potential measurement

The Polyacrylamide gels are synthesised using the methodology detailed in the step by step methodology in the supplemental information in Methods S3 ″Polyacrylamide Hydrogel Synthesis Procedure”. The Polyacrylamide gels are suspended in a sodium chloride solution (0.08%) between aluminum electrodes (aluminum electrodes gave the best results of bending with minimal corrosion) as shown in Figure 1 section a and b. The solution increases the ionic concentration difference between the ionic hydrogel and surrounding solution, allowing bending because of the change in the absorption property of the gel.^77^ A camera records bending motion which is translated into angle values. A black screen is placed behind the hydrogels to increase visibility on recorded video data, against a black background the gels appears blue because of Rayleigh and Mie scattering.^78^ Sequences of voltages are used to stimulate the gel (+31 V referred to as 1 and -31 V referred to as −1, across the 6.4 mm width of the gel), 31 V was used as it was the maximum output of the power supply and caused swelling in the gel at a reliable rate during experimentation. AINA219 current sensor is placed in line with the driving voltage, recording current and voltage potential values with time stamps to align with the video data. This voltage potential measurement system does introduce some noise but does not effect the collected results.^79^ Eight stimulation sequences are applied to the gel representing every combination of positive and negative stimulations in a 3-input sequence (-1-1-1, -1-11, -11-1, −111, 1-1-1, 1–11, 11-1, 111).

• For each experiment fresh aluminum electrodes were applied to the suspension apparatus with wires affixed behind the electrodes.
• The gel was placed between electrodes with both sides touching the gel shown in Figure 1 section a and b. A voltage of 31v was applied in both polarities twice for 10 s each time to adhere the gel to the electrodes by pulling the charged gel polymer into the texture of the electrode. 10 s was found to be the shortest time needed to adhere the electrodes.
• The suspension apparatus along with the gel were placed in a beaker of sodium chloride solution shown in Figure 1 section a.
• The camera was adjusted to place the gel in center view parallel to the suspension apparatus.
• The video recording was started along with the voltage potential recording. A sequence of stimulations were applied to the gel, each stimulation applied for 90 s with 10 s breaks between stimulations. A stimulation length of 90 s was used being the shortest time allowing visible bending to occur.
• The video was segmented into a set of pictures representing the output of each stimulation, with an initial image used as the gel at t = 0.

The code used to analyze the data collected from these experiments can be found in the repository 10.5281/zenodo.7274655 in folder “Experiment _1-Memory _Mechanics _Through _Ion _Migration _and _Voltage _Potential _Measurement”.In addition to these steps temperature and humidity can affect the response of EAP hydrogels. The elastic properties of the gel are a function of temperature.^21^^,^^47^ For this reason, the purified water used in the ionic solution, although not temperature controlled during the experiment, is from a temperature controlled source (22C), so the experiments would always start at the same temperature. As the gels rely on water molecules to swell the hydrogel’s water content also affects the response and as such so can humidity. Suspension in the ionic solution prevents the influence of ambient humidity on the gels during experimentation. The gels are also stored in watertight containers in between experimentation to maintain moisture content until use.

#### Applying EAP gel to a probabilistic moore machine automata

The input sentences are applied to the gel using the same sequential stimulation procedure and apparatus as in the Memory Mechanics experiment of the previous section. Every permutation of input symbols (8 sequences) is applied to a new gel so that sequences have no influence on each-other. This is because there is no guarantee that, in practice, previous stimulation on a gel will not affect future behavior even after significant rest time. This is done 8 times resulting in 64 separate experiments to collect enough data to establish results consistency in every path through a 3-symbol tree. These input sequences are applied with the same voltage and timing as in the Memory Mechanics experiment, using 90 s per input with 10 s breaks to change electrode polarity. Values used for concentration, voltage, and times can be altered changing the gel’s response to input sequences but requires more detailed experimentation to analyze the best conditions, beyond the scope of this study. Once data is collected the video is segmented into a series of images, representing the end of each input symbol and one at t = 0 used to find the change in bending angle. As bending occurs over the length of the gel the angle is measured using the mid-point at the gel’s tip and the edge of the suspension apparatus, shown in Figure 1 section C. The code used to analyze the data collected from these experiments can be found in the repository https://doi.org/10.5281/zenodo.7274655 in folders ”Experiment _2-Applying _EAP _gel _to _a _Moore _Machine _Automata” and “Experiment _3-Applying _Moore _Reservoir _Hybrid _to _Collected _Data”.

### Quantification and statistical analysis

All data analysis was performed using code written in the MATLAB environment, details on this code and MATLAB datasets can be found in the readme in the code and dataset repository as indicated by the DOI in the key resources table. When collecting the data, each path through the 3 layer automaton was explored with a new gel, this led to 8 gels required to fully explore every path of the automaton state tree. Each path was then repeated a further 8 times to ensure accurate representation in the results, this gave a total of 64 individual experiments ran with a new gel for each experiment. The statistical analysis, of the application of the EAP hydrogels to reservoir computing, is detailed in section “probabilistic mooreautomata as a computational reservoir”. Polynomials of varying degrees were applied to the resultant reservoir of each threshold pair to allow the comparison of memory usage, where the degree of the polynomial represents the memory needed by the computer to replicate the computation in the EAP gel reservoir. The significance in this application of EAP gels to computation was justified if the polynomial representation required as much memory as the resultant reservoir, thus making the EAP reservoir more efficient.

## Data Availability

• Datasets containing the voltage potential responses of the gel and automaton angle data respectively have been deposited at Zenodo and are publicly available as of the date of publication. DOIs are listed in the key resources table. • All original code has been deposited at Zenodo and is publicly available as of the date of publication. DOIs are listed in the key resources table. • Any additional information required to reanalyze the data reported in this paper is available from the lead contact upon request.
