# Supplementary material for: Electroactive polymer gels as probabilistic reservoir automata for computation
Source: iScience. 2022 Nov 14;25(12):105558. doi: 10.1016/j.isci.2022.105558 (PMC9708913; doi:10.1016/j.isci.2022.105558)
Supplement: Document S1. Figures S1–S4, Table S1, and Methods S1–S3 [file mmc1.pdf]

iScience, Volume *25*

Supplemental information

Electroactive polymer gels as probabilistic reservoir automata for computation

Vincent Strong, William Holderbaum, and Yoshikatsu Hayashi

Methods S1 Post-Processing Experimental Results to Reduce Variance

Referenced in the main text under section ”Applying EAP gel to a Probabilistic Moore Machine Automata”, and related to Figure 4 in the main text. To effectively judge consistency some post-processing is needed to filter noise caused by unavoidable inconsistencies between experiments e.g. positioning of gel, gel surface texture, slight synthesis differences between batches etc. To filter noise in the current setup a gain and offset parameter are used.

$$\boldsymbol{\Theta}_i = \alpha\theta_i - \theta_0 \tag{1}$$

$$\boldsymbol{\Theta} = (\boldsymbol{\Theta}_1, \boldsymbol{\Theta}_2, \boldsymbol{\Theta}_3)\ \{\boldsymbol{\Theta}_i \in \mathbb{R}\} \tag{2}$$

$$\text{Where } \alpha = \underset{a\in\mathbb{R}}{\text{arg min}} \sum_{i=1}^{N} (a\theta_i - \bar{\theta_{\boldsymbol{I}}})^2 \tag{3}$$

Where $\bar{\theta_{\boldsymbol{I}}}$ is the average output angle in the collected data for input sequence $\boldsymbol{I}$

The offset parameter accounts for differences in surface texture that cause initial bending in the gel at t=0 [1], by subtracting the bending angle at t=0 from further angles all gels have the same zero reference, as shown in equation 1. Where $\boldsymbol{\Theta}$ is the vector of processed angles as defined in equation 2, α is a gain parameter, θ_i is the angle after input symbol i, θ_0 is the bending angle from the initial posture of the gel at t=0.

The gain parameter is used to reduce the effect of differences in synthesis between batches. Changing the chemical ratio changes swelling rate due to change in elasticity [2]. A gain parameter is applied to each gel to minimises its distance from the average output angle for that input sequence. This is shown in equation 3 where $\bar{\theta_{\boldsymbol{I}}}$ is the average output angle in the collected data for input sequence $\boldsymbol{I}$. As only one gain and offset parameter is used per gel this method compensates for procedural flaws without forcing data into a fabricated pattern.

The variance [3] in output angles for each input sequence is used to evaluate the success of this post processing. Table S1 shows variance for each input sequence $\boldsymbol{I}$, before and after the gain parameter along with the reduction, giving an average reduction of 33.5 degrees^2. This shows notable improvement, mitigating a significant portion of noise from the experimental procedure.

Methods S2 Optimisation of Output Encoding Thresholds to Maximize Automaton Response

Referenced in the main text under section ”Applying EAP gel to a Probabilistic Moore Machine Automata” and Figure 5. The selection of output thresholds alter the response of the gel as an automaton. The bending angle is converted to a symbol as shown in equation 4 where; $\boldsymbol{O}$ is the output symbol, $\boldsymbol{\Theta}$ is the processed output angle. t_1 and t_2 are thresholds 1 and 2 respectively which remain constant for a given Moore machine. By altering threshold values and analysing the resultant probabilistic Moore machine the gel automaton's range of capability can be found. The ideal Moore machine would maximize certain evaluation criteria;

- Maximize predictability: Achieved by maximizing the probability that each $\boldsymbol{I}$ vector will consistently result in the same $\boldsymbol{O}$ vector between activations of the probabilistic Moore machine.

- Maximize computational range: Achieved by realising equal distribution of output symbols in the $\boldsymbol{O}$ vectors generated by the probabilistic Moore machine.
- Maximize computational versatility: Achieved by maximizing the number of unique $\boldsymbol{O}$ vectors given by the probabilistic Moore machine across all $\boldsymbol{I}$ vectors.

Altering the threshold values affects these criteria in the generated probabilistic Moore machines, thus every combination of 2 thresholds are used to generate graphs representing each criteria. The range of threshold values tested are bound by the maximum and minimum recorded bending angles (-31.3 and 29.3) with a step of 0.5 degrees ($\boldsymbol{\Theta}_{min}, \boldsymbol{\Theta}_{min}+0.5, ..., \boldsymbol{\Theta}_{max}-0.5, \boldsymbol{\Theta}_{max}$). The results of this can be seen in figure S3 of the supplementary document and show how thresholds affect each criteria.

$$\boldsymbol{O} = \omega\left(\boldsymbol{\Theta}, t_1, t_2\right) = \left\{\begin{matrix} -1 & \boldsymbol{\Theta} < t_1 \\ 0 & t_1 \leq \boldsymbol{\Theta} \leq t_2 \\ 1 & \boldsymbol{\Theta} > t_2 \end{matrix}\right\} \quad (4)$$

Finding a value that fully maximises all these criteria is impossible as the number of unique output sequences increases inversely to the probability and standard deviation shown in figure S3 A, B and C. The sum of squared errors is used to simplify this multi-objective optimisation into a single function to be minimized. The error graph, figure S3 D, shows the threshold values that achieve the most significant ability from the probabilistic Moore machine at the minima of the graph. For data collected these thresholds were found to be -7.8 and 2.7 for threshold 1 and 2 respectively.

Methods S3 Polyacrylamide Hydrogel Synthesis Procedure

Referenced in the main text STAR Methods under section Method Details. This section details the procedure used to synthesise and mould the polyacrylamide hydrogels for use in the experiments. Polyacrylamide gels were prepared by free-radical polymerisation [4], using a procedure derived from the paper ”Collapse of gels in an electric field” [5]. The detailed synthesis procedure is numbered below.

1. Place 100ml of distilled water in a flask, cover the top with plastic with small holes in it to let air escape during degassing.
2. Place some ice in a vacuum chamber then place flask inside chamber, pressurise and leave for 20 minutes to degas.
3. Into a separate flask and measure the following using an analytical balance within a fume hood:
    - Acrylamide (5g), the linear constituent
    - N, N'- methylenebisacrylamide (0.133g), the tetrafunctional crosslinking constituent
    - ammonium persulfate (40mg), the initiator
4. Once degassing has finished pour distilled water into flask containing chemicals, place in ice box and mix for 20 minutes or until no more of the particles are dissolving.

5. Prepare the mould for moulding the gel into a small strips (rectangular cuboid 10mm X 30mm X 10mm).
6. Using a pipette measure 240μl of TEMED into the flask containing the dissolved chemicals (N, N, N, N-tetramethylethylenediamine (TEMED), the accelerator).
7. Using a serological pipette place the solution into each of the mould sections until each is completely filled. Place remaining solution into beaker as a reference sample to check if the solution has gelled properly.
8. The solution will start to gel in 5 minutes and completely gel in 5 hours.
9. Once all of chemicals have polymerised pour small amount of water over gels to loosen from mould.
10. Place gels into distilled water to equalize for 24 hours.
11. Fill a beaker to a significant enough level to completely submerge the gel strips, add 1.2% TEMED using a serological pipette, mix the solution until dispersed.
12. Place the gel strips in the beaker containing the TEMED solution to undergo hydrolysis, leave for a week to ionise.
13. Pour off TEMED solution, then fill beaker with distilled water to wash away any residual TEMED, pour away water.
14. Place gels strips into plastic sealed container to be transferred to experiment.

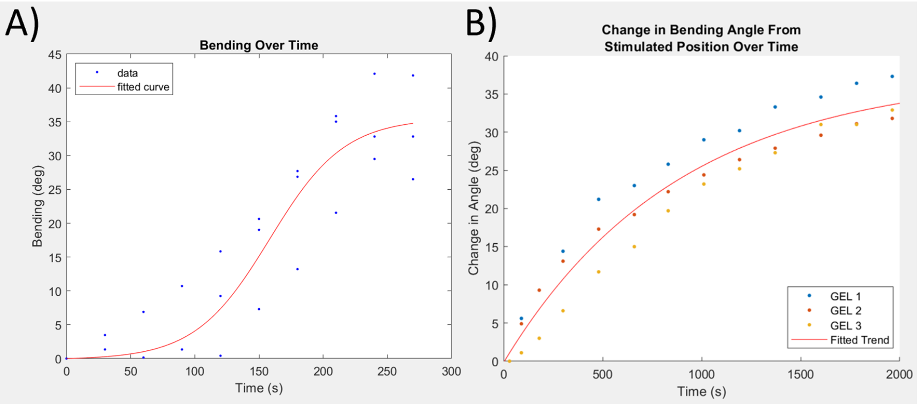

Figure S1: Gel bending over time with and without stimulation. Related to Figure 3 in the main text. **A)** The trend of the EAP hydrogel bending over time, taking approximately 4 minutes to fully bend with electrical stimulation of 31v. **b)** The trend of the EAP hydrogel bending from a fully stimulated position over time with no stimulation, measured as a difference from fully stimulated position taking significantly longer to de-swell than swell.

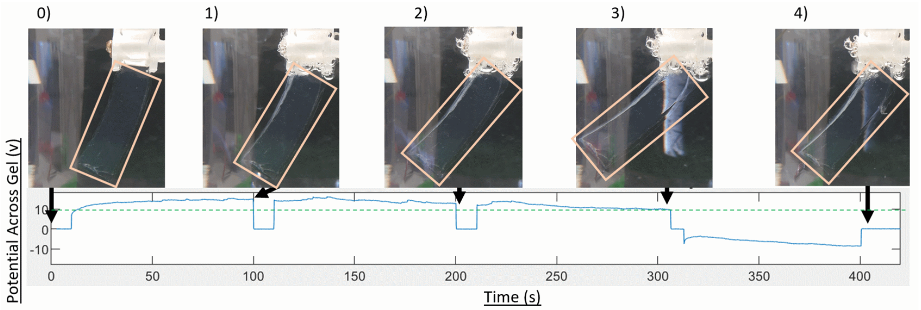

Figure S2: Voltage potential across gel over time. Related to Figure 2 in the main text. Key frames from recorded video illustrate bending at t=0, t=100, t=200, t=300 and t=400 and labelled as 0,1,2,3,4 respectively. The green dashed line is used to compare the voltage potential at end of a stimulation with that at beginning. The sequence applied in the experiment show is -1,-1,-1,1

Table S1: Comparison of variance of angles for different input sequences ($\boldsymbol{I}$) with and without gain parameter. Related to methods S2 ”Post-Processing Experimental Results to Reduce Variance”, Equations 1, 2, and 3 in the supplementary text, and related to section ”Applying EAP gel to a Probabilistic Moore Machine Automata” and Figure 4 in the main text.

$\boldsymbol{I}$	**Variance No Gain**	**Variance With Gain**	**Reduction**
-1	35.28	14.81	20.48
1	30.92	21.27	9.65
-1,-1	30.45	10.95	19.50
-1,1	32.48	16.53	15.95
1,-1	23.30	8.71	14.59
-1,-1	57.73	26.51	31.22
-1,-1,-1	52.80	59.83	-7.03
-1,-1,1	161.57	85.23	76.35
-1,1,-1	33.05	15.84	17.21
-1,1,1	89.83	7.34	82.49
1,-1,-1	59.02	2.83	56.19
1,-1,1	5.02	4.25	0.76
1,1,-1	88.48	54.95	33.53
1,1,1	103.57	6.15	97.42

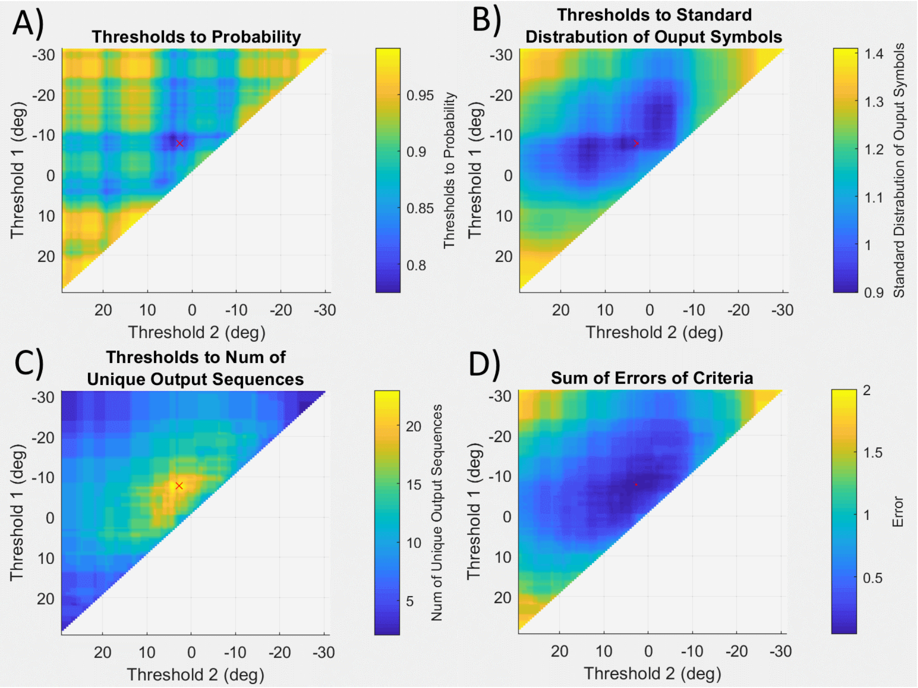

Figure S3: Error results of criteria used to measure the affect of thresholds as described in the section titled Threshold Optimisation. Related to method S3 "Optimisation of Output Encoding Thresholds to Maximize Automaton Response" in the supplementary text, and related to section "Applying EAP gel to a Probabilistic Moore Machine Automata" and Figure 5 in the main text. The optimised thresholds are shown by the red cross on each plot, found to be -7.8 & 2.7 for thresholds 1 (t_1) & 2 (t_2) respectively. **A)** Maximize predictability, **B)** Maximise computational range, **C)** Maximize computational versatility, **D)** Sum of errors of all evaluation criteria to indicate best threshold selection.

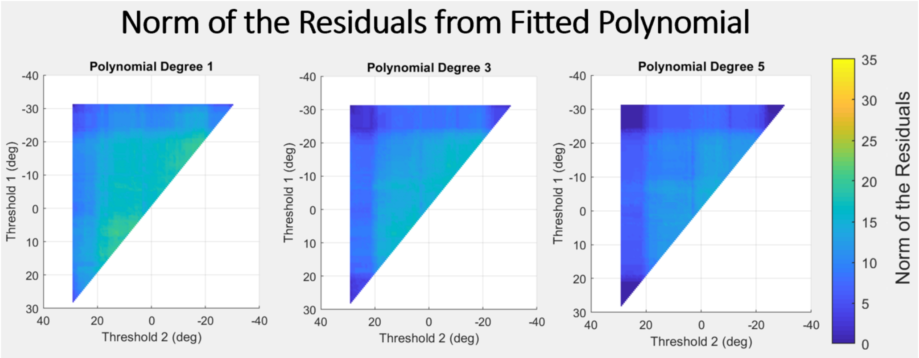

Figure S4: Heat map for the norm of residuals error of fitted polynomials, against the threshold values used in the output layer of the PMA reservoir instance that the polynomial is fitted to. Related to Figure 7 in the main text. Each pair of threshold values is used to generate a PMA reservoir by being applied to the output layer and a polynomial is fitted to the mapping function of these reservoirs. These graphs show the results of polynomial fitting for polynomial degrees 1, 3 and 5. The colour bar shows the relative norm of residual values

References

[1] MNI Shiblee, K Ahmed, M Kawakami, H Furukawa, (2019). 4d printing of shape-memory hydrogels for soft-robotic functions. *Advanced Materials Technologies* **4**, 1900071.

[2] AK Denisin, BL Pruitt, (2016). Tuning the range of polyacrylamide gel stiffness for mechanobiology applications. *ACS applied materials & interfaces* **8**, 21893–21902.

[3] Y Zhang, H Wu, L Cheng, (2012). Some new deformation formulas about variance and covariance in *2012 Proceedings of International Conference on Modelling, Identification and Control*. (IEEE), pp. 987–992.

[4] T Tanaka, et al., (1980). Phase transitions in ionic gels. *Physical Review Letters* **45**, 1636.

[5] T Tanaka, I Nishio, ST Sun, S Ueno-Nishio, (1982). Collapse of gels in an electric field. *Science* **218**, 467–469.
